# Color stability, surface roughness, and surface morphology of universal composites

**DOI:** 10.1007/s10266-025-01108-2

**Published:** 2025-04-28

**Authors:** Mohammad Meniawi, Nazlı Şirinsükan, Esra Can

**Affiliations:** Faculty of Dentistry, Department of Restorative Dentistry, Yeditepe University, Bağdat Caddesi No: 238, Göztepe, Istanbul, Turkey

**Keywords:** Color stability, Surface roughness, Universal composites, Highly filled flowable composite, Injectable composite

## Abstract

This study aimed to evaluate the surface roughness (Ra; µm), color stability (∆E_00_), and surface morphology of universal composites after exposure to discoloring solutions. 200 composite discs (2 × 10 mm) were prepared using four paste type (Estelite Asteria, G-aenial A’chord, Filtek Universal, and Filtek Ultimate) and one highly filled flowable (G-aenial Universal Injectable) universal composites (n = 40). Following storage in distilled water, each composite was randomly divided into four subgroups according to the discoloring solutions; black tea, coffee, red wine, and artificial saliva. Baseline, 7, 14, and 21-day color measurements (∆E_00_) were performed while Ra was evaluated at baseline and 21 days. Data were analyzed using three-way ANOVA and post-hoc Tukey’s tests (*p* < 0.05). The correlation between ∆E_00_ and Ra was evaluated using Pearson’s correlation test (*p* < 0.05). ∆E_00_ and Ra were significantly influenced by the composite (*p* < 0.001), discoloring solution (*p* < 0.001), and the evaluation period (*p* < 0.001) while significant interactions were evaluated between the variables for both test methods. For all the composites and evaluation periods, red wine resulted significantly in the highest color change which was followed by black tea and coffee (*p* < 0.001). All the composites revealed significantly higher Ra after exposure to discoloring solutions (*p* < 0.001). There was a positive correlation between ∆E_00_ and Ra with all the tested universal composites (*p* = 0.0001). All the tested universal composites revealed clinically unacceptable color change and increased surface roughness after exposure to discoloring solutions. Highly filled flowable universal composite showed the highest color stability in coffee, red wine, and black tea.

## Introduction

Due to advancements in both mechanical and aesthetic qualities, resin composites have become one of the most widely used restorative materials in dental practice. Manufacturers are now producing a variety of universal composites that can meet all the requirements of the anterior and posterior regions, including high wear and fracture resistance, color stability, and gloss retention, all of which influence the longevity of restorations [1]. The type of the resin matrix, amount of filler particles, adsorption and absorption of stains, type of staining solution, and chemical interactions between the composite and stains are factors that affect the staining capacity of the resin composites [2]. However, several studies have demonstrated that all types of resin composites, including universal composites, are vulnerable to discoloration when exposed to the oral environment for extended periods [3, 4]. This phenomenon may occur due to intrinsic or extrinsic variables [5–7]. Intrinsic discoloration is caused by alterations in the chemical structure of the resin composites under various physical and chemical conditions. Inadequate polymerization [8], type of initiator [9, 10], type of resin matrix, inorganic fillers, and alteration of the silane coupling agent may result in permanent discoloration of the subsurface and internal layers of the resin composites [11]. On the other hand, extrinsic factors causing surface discoloration are chromogenic chemicals that adsorb or absorb to the surface or subsurface and can be removed mechanically or chemically [5, 12–14] Patients’ eating and drinking habits also play an essential role in the resistance of composite restorations to discoloration [15, 16]. Various beverages, such as coffee [17, 18], black tea [6], wine [18, 19], alcoholic beverages [12, 20], carbonated drinks [17, 21], and fruit juices [22] have been found to cause significant extrinsic discoloration in resin composites.

Smooth surfaces and margins of composite restorations reduce the risk of biofilm adhesion and maturation, recurrent caries, gingival irritation, and discoloration, and an increase in surface roughness may be one of the reasons for external discoloration [23, 24]. It has been well documented in the literature that similar to the discoloration phenomenon, the surface roughness of composite restorations is also closely related to the structural properties of the composites, susceptibility to water sorption, and proper finishing and polishing procedures [14, 25–27]. The influence of polishing on the color stability of resin composites can be linked to the surface topography of the material after polishing. Rougher surfaces are more prone to extrinsic discoloration [28]. Therefore, this *in-vitro* study aimed to evaluate the color stability, surface roughness (Ra; µm), and surface morphology of the universal composites; supra nano-fill Estelite Asteria (Tokuyama, Japan), nano-hybrid G-aenial A’chord (GC, Japan), highly filled flowable nano-fill G-aenial Universal Injectable (GC, Japan), nano-fill Filtek Universal (3 M, USA) and nano-fill Filtek Ultimate (3 M, USA) after exposure to coffee, black tea, red wine, and artificial saliva over a period of 21-days. The tested null hypotheses were as follows: (1) There would be no significant differences in the color stability of each composite in coffee, black tea, red wine, or artificial saliva at baseline and at 7, 14, and 21-days. (2) There would be no significant differences in the color stability among the different universal composites in each discoloring solution at baseline and at 7, 14, and 21-days. (3) There would be no significant differences in the surface roughness of each composite at baseline and at 21-days in coffee, black tea, red wine, and artificial saliva. (4) There would be no significant differences in surface roughness among the different universal composites in each discoloring solution at baseline and at 7, 14, and 21-days.

## Materials and methods

The sample size was calculated based on the estimated effect size between groups, according to the literature for contact profilometry [29] and color stability [30, 31], and it was determined that 10 specimens were needed for each group to achieve a medium effect size with 95% power and a 5% type 1 error rate. A total of 200 specimens, 40 of each type of composite, supra nano-fill Estelite Asteria (Tokuyama, Japan), nano-hybrid G-aenial A’chord (GC, Japan), nano-fill G-aenial Universal Injectable (GC, Japan), nano-fill Filtek Universal (3 M, USA), and nano-fill Filtek Ultimate (3 M, USA) in A1 shade, 10 mm in diameter and 2 mm in thickness, were prepared using metallic molds. Each material was inserted into the cylindrical metal mold and pressed between two opposing Mylar matrices (Hawe Stopstrips; Kerr CA, USA), which were then covered with a glass slide (1 mm thick) to extrude excess material and produce a smooth, flat surface. The specimens were then polymerized on a glass slide at a light intensity of 1200 mW/cm^2^ (Bluephase N; Ivoclar, USA) from the upper and bottom surfaces. The compositions and manufacturers of the materials are listed in Table 1.

**Table 1:** The compositions and manufacturers of the materials used in the study

Universal composites	Type	Manufacturer	Resin matrix	Filler type	Filler load (%)	Lot number
Estelite asteria	Supra nano-fill universal composite	Tokuyama, Tokyo, Japan	Bis-GMA, Bis-MPEPP, TEGDMA, UDMA	Supra-nano spherical filler (200 nm silica-zirconia), composite filler (200 nm silica-zirconia)	82% wt 71% vol	185EY2
G-aenial A’chord	Nano- hybrid universal composite	GC Europe N.V: Leuven, Belgium	Bis-MPEPP, UDMA	300 nm barium glass filler, 16 nm fumed silica, organic filler (300 nm barium glass; 16 nm fumed silica)	82% wt 65% vol	220524A
G-aenial injectable	Nano- hybrid universal composite	GC Europe N.V: Leuven, Belgium	UDMA, Bis-MPEPP, TEGDMA	150 nm barium glass fillers	69% wt 50% vol	2208161
Filtek universal	Nano-fill universal composite	3 M St.Paul, MN United States	AUDMA, AFM, diurethane-DM, 1,12-dodecane-DMA	20 nm silica filler,4 to 11 nm zirconia filler, zirconia/silica cluster filler (composed of 20 nm silica and 4 to 11 nm zirconia particles), and 100 nm ytterbium trifluoride filler	76.5% wt 58.4% vol	9406531
Filtek ultimate	Nano-fill universal composite	3 M St.Paul, MN United States	UDMA, Bis-GMA, PEGDMA, Bis-EMA, TEGDMA	20 nm silica filler, 4 to 11 nm zirconia filler, zirconia/silica cluster filler (composed of 20 nm silica and 4 to 11 nm zirconia particles), average cluster particle size of 0.6 to 10 microns	78.5% wt 63.3% vol	3920A1B

After storage in distilled water for 24 h at 37 °C, the specimen’s upper and bottom surfaces were wet finished with 600 grit SIC paper (Phoenix Beta; Zimpara, Kocaeli, Turkey) at 6000 Rpm. Then, all specimens were wet polished on both sides with two-step polishing wheels (Twist DIA; Kuraray, Japan) at 10,000 Rpm for 20 s each, and the polishing wheels were changed after every three specimens. Final polishing of the specimens was performed with 1 µm, and 0.5 µm grit diamond pastes (Diamond polish; Ultradent; USA) using Super-snap Super buff cups (Shofu; Japan,) for 20 s each at 5000 Rpm. All the specimens were then washed with copious amounts of water and dried. Each composite group was then randomly divided into four subgroups according to the discoloring agent: coffee, black tea, wine, and artificial saliva (n = 10) and placed in individual test tubes filled with distilled water for 24 h. After 24 h, the specimen’s baseline color (spectrophotometer; Minolta CM-2600d) and surface roughness (profilometer; Perthometer M1 Mahr, Germany) were measured from the opposite sides of the specimens. The second operator, who was blind to the type of the composite, performed all of the color and roughness evaluations.

Following baseline measurements, each specimen was immersed in 200 mL of freshly prepared coffee (Nestle Nescafe black instant coffee in individual bags), black tea (Lipton Black tea bags), and wine (Villa Doluca Bogazkere) for 6 h daily, rinsed with distilled water, and lightly brushed with a soft toothbrush for 5 s, and then stored in artificial saliva for 18 h at a temperature of 37 °C. Artificial saliva served as the control and the control group specimens from each composite type were stored in artificial saliva for 24 h. The black tea solution was prepared by placing five Lipton Black tea bags in boiling distilled water (200 mL). The test tubes containing the specimens were filled with black tea, and the specimens were stored for 6 h. This process was repeated every 24 h, and new black tea was prepared daily to prevent any possible chemical changes and precipitation. For coffee, the solution was prepared by pouring five instant coffee bags into 200 mL water, and the same procedure was followed as that for the black tea groups. For red wine, the specimens were placed in 200 mL wine at room temperature for 6 h, and the solution was renewed each day. All test tubes were numbered and labeled according to the universal composite, discoloring agent, and evaluation period. The immersion protocol for discoloring agents was repeated for 21-days. A flowchart of the study is presented in Table 2.

**Table 2:** Flowchart of the study

Materials	Groups	Staining regimen
1.Supra nano-fill composite Estelite Asteria (Tokuyama, Japan) (n = 40) 2.Nano-hybrid composite G-aenial A’chord (GC, Japan) (n = 40) 3.Nano-hybrid composite G-ænial Universal Injectable (GC. Japan) (n = 40) 4.Nano-fill composite Filtek Universal (3 M, USA) (n = 40) 5.Nano-fill composite Filtek Ultimate (3 M, USA) (n = 40)	Artificial saliva (n = 40)	Baseline color measurement + immersion in artificial saliva, color measurement after 7-14-21 days,
	Coffee (n = 40)	Baseline color measurement + immersion in coffee for 6 h and artificial saliva for 18 h, color measurement after 7-14-21 days
	Black tea (n = 40)	Baseline color measurement + immersion in black tea for 6 h and artificial saliva for 18 h, color measurement after 7-14-21 days
	Red Wine (n = 40)	Baseline color measurement + immersion in red wine for 6 h and artificial saliva for 18 h, color measurement after 7-14-21 days

### Color evaluations

After 24 h and at 7, 14, and 21 days, the specimens were removed from the test tubes, washed for 30 s with tap water, brushed for 5 s with a soft brush, and dried using absorbent paper. The samples were then placed in a spectrophotometer (Minolta CM-2600d; Konica Minolta, Munich, Germany), and readings from the midpoint of each specimen were recorded. Color change (∆E_00_) was calculated using the CIEDE2000 color evaluation system at each evaluation period. The color changes between the time points were calculated and expressed as 24-h ΔE_00_ (between baseline and 24-h), 7-days ΔE_00_ (between baseline and 7 days), 14-days ΔE_00_ (between baseline and 14-days) and 21-days ΔE_00_ (between baseline and 21-days). The following equation was used for the calculation, in which ΔL’, ΔC’ and ΔH’ represent luminosity, chroma and hue respectively, RT refers to the interaction between chroma and hue in the blue region, weighting functions SL, SC, and SH are used to adjust the overall color difference in L*, a*, and b* coordinates and KL, KC and KH are correction terms for experimental conditions.

$${\text{The }}\Delta {\text{E}}_{00} {\text{color difference formula}}:\sqrt {\left[ {\left( {\frac{\Delta L}{{K_{L} S_{L} }}} \right)^{2} + \left( {\frac{\Delta C}{{K_{C} S_{C} }}} \right)^{2} + \left( {\frac{\Delta H}{{K_{H} S_{H} }}} \right) + R_{T} \left( {\frac{\Delta C}{{K_{C} S_{C} }}} \right)\left( {\frac{\Delta H}{{K_{H} S_{H} }}} \right)} \right]}$$

### Surface roughness evaluation

The disc-shaped specimens’ surface roughness (Ra; µm) at baseline and at the end of 21-days was evaluated with a profilometer (Perthometer M1 Mahr, Germany). For each composite specimen, on the opposite side of the specimens where the color evaluations were performed, three measurements were recorded at three different locations with a cut-off length of 0.25 mm and a tracing length of 0.8 mm at a stylus speed of 0.1 mm/s. The roughness value (Ra; µm) was calculated as the average of these three readings. The profilometer was periodically calibrated during the experimental period.

### Scanning electron microscopy (SEM) evaluation

One specimen that had the closest to the mean roughness value from each group was selected and cleaned with an ultrasonic cleaner, coated with platinum, and observed at × 2000 magnification at baseline and 21-days for SEM evaluation (JSM-6400 SEM, JEOL, Tokyo, Japan).

### Statistical analysis

The influence of the composite type, discoloring solution, and evaluation period on color stability (∆E_00_) and surface roughness (Ra) of universal composites was statistically analyzed using three-way ANOVA and post-hoc Tukey’s tests at a significance level of 0.05 (SPSS Version 28.0; Chicago, IL, USA). The correlation between the color stability and surface roughness was evaluated using Pearson’s correlation test.

## Results

According to the results of three-way ANOVA and post-hoc Tukey’s tests, ∆E_00_ and Ra were significantly influenced by the composite (*p* < 0.001), discoloring solution (*p* < 0.001), and evaluation period (*p* < 0.001), and significant interactions were found between the variables. Surface roughness and color stability showed a statistically significant positive correlation when all composites and discoloring agents were assessed together (*r* = *0.655, p* = 0.0001).

### ***Color evaluation (***∆E_00_***)***

The mean ∆E_00_ values of the tested universal composites stored in different discoloring solutions at 7, 14, and 21-days are presented in Table 3. ∆E_00_ was significantly affected by the type of composite, discoloring solution, and evaluation period (*p* < 0.001). The interaction between composites, discoloring agents, and evaluation periods was statistically significant (*p* < 0.001). All universal composites presented significantly higher color changes at 7, 14, and 21-days compared to the baseline for each discoloring agent and artificial saliva (*p* < 0.001).

**Table 3:** Mean ∆E_00_ values (± standard deviations) and Statistical Analysis of the Tested Universal Composites at 7, 14 and 21 days

Discoloring solutions	Filtek ultimate	Filtek universal	G-aenial A’chord	G-aenial injectable	Estelite asteria
Mean ∆E_00_ values (± standard deviations) and statistical analysis of the tested universal composites at 7 days					
Artificial saliva	0.642 ± 0.136^aA^	0.737 ± 0.152^aA^	0.695 ± 0.096^aA^	0.909 ± 0.153^aA^	0.78 ± 0.065^aA^
Coffee	2.434 ± 0.208^bA^	2.619 ± 0.458^bA^	2.84 ± 0.634^bA^	2.632 ± 0.198^bA^	2.417 ± 0.240^bA^
Black tea	2.451 ± 0.286^bA^	2.762 ± 0.372^bA^	3.29 ± 0.238^bB^	2.901 ± 0.355^bAB^	2.779 ± 0.371^bA^
Wine	4.474 ± 0.376^cA^	3.743 ± 0.398^cB^	5.699 ± 0.298^cC^	3.792 ± 0.451^cB^	5.478 ± 0.225^cC^
Mean ∆E_00_ values (± standard deviations) and statistical analysis of the tested universal composites at 14 days					
Artificial saliva	0.794 ± 0.157^aA^	0.993 ± 0.127^aA^	1.03 ± 0.113^aA^	1.104 ± 0.139^aA^	1.119 ± 0.137^aA^
Coffee	3.252 ± 0.722^bA^	3.523 ± 0.294^bA^	3.91 ± 0.351^bA^	3.62 ± 0.498^bA^	3.789 ± 0.555^bA^
Black tea	5.735 ± 0.369^cAB^	5.560 ± 0.459^cA^	6.164 ± 0.734^cAB^	6.114 ± 0.636^cAB^	6.41 ± 0.481^cB^
Wine	5.989 ± 0.502^cC^	6.218 ± 0.536^cBC^	6.714 ± 0.312^cB^	6.137 ± 0.393^cBC^	8.06 ± 0.488^dA^
Mean ∆E_00_ values (± standard deviations) and statistical analysis of the tested universal composites at 21 days					
Artificial saliva	1.035 ± 0.165^aA^	1.19 ± 0.132^aA^	1.361 ± 0.280^aA^	1.426 ± 0.110^aA^	1.286 ± 0.232^aA^
Coffee	6.645 ± 0.506^bA^	5.924 ± 0.482^bB^	6.215 ± 0.636^bAB^	5.419 ± 0.706^bC^	6.753 ± 0.546^bA^
Black tea	10.534 ± 0.349^cBC^	11.251 ± 1.969^cAC^	12.03 ± 0.710^cA^	9.468 ± 1.148^cB^	11.634 ± 1.049^cAC^
Wine	13.131 ± 0.663^dAB^	16.382 ± 0.927^dC^	14.32 ± 0.395^dA^	12.887 ± 0.972^ dB^	14.238 ± 0.975^dA^

Different capital superscript letters indicate significant differences between different universal composites for each discoloring solution while different superscript letters indicate differences between discoloring solutions within each composite (*p* < 0.05)

At 7-days of storage in artificial saliva and coffee, there were no significant differences between the tested composites (*p* > 0.05)*.* In black tea, the rank of ∆E_00_ values was G-aenial A’chord = G-aenial Injectable > Filtek Ultimate = Filtek Universal = Estelite Asteria, whereas in wine, ∆E00 values were Estelite Asteria = G-aenial A’chord > Filtek Ultimate > Filtek Universal = G-aenial Injectable.

At 14-days of storage in artificial saliva and coffee, there were no significant differences between ∆E_00_ values of the tested composites (*p* > 0.05). In black tea, ∆E_00_ values were Estelite Asteria > G-aenial Injectable = G-aenial A’chord = Filtek Ultimate > Filtek Universal. In wine, ∆E_00_ values were Estelite Asteria > G-aenial A’chord = Filtek Universal = G-aenial Injectable and G-aenial A’chord > Filtek Ultimate.

At 21-days, there were no significant differences between the tested composites in artificial saliva *(p* > *0.05).* For coffee, Filtek Ultimate = Estelite Asteria > Filtek Universal > G-aenial Injectable. For black tea, G-aenial A’chord > G-aenial Injectable = Filtek Ultimate and Filtek Universal = G-aenial Accord = Estelite Asteria. For wine, Filtek Universal > Estelite Asteria = G-aenial A’chord = Filtek Ultimate > G-aenial Injectable.

For discoloring solutions, storage in artificial saliva resulted in the lowest ∆E_00_ in each composite at each evaluation period (*p* < 0.001), while there were no significant differences between coffee and black tea at 7-days (*p* > 0.05); however, black tea resulted in a significantly higher color change than coffee for each composite at the 14- and 21-day evaluation periods (*p* < 0.001). Wine resulted in the highest color change for all composites at 7-and 21-days of evaluation (*p* < 0.001).

### Surface roughness (Ra) evaluation

Baseline and 21-day Ra values of the universal composites tested with different discoloring agents are presented in Table 4. All the universal composites presented significantly higher Ra at 21-days compared to the baseline for each discoloring agent and artificial saliva (*p* < 0.001).

**Table 4:** Mean Ra values (± standard deviations) and Statistical analysis of the tested universal composites at baseline and 21 days

Discoloring solutions	Filtek ultimate	Filtek universal	G-aenial A’chord	G-aenial injectable	Estelite asteria
Mean Ra values (± standard deviations) and statistical analysis of the tested universal composites at baseline					
Artificial saliva	0.0293 ± 0.0024	0.0306 ± 0.0025	0.0323 ± 0.0022	0.0310 ± 0.0028	0.0321 ± 0.0026
Coffee	0.0302 ± 0.0029	0.0312 ± 0.0027	0.0315 ± 0.0030	0.0306 ± 0.0026	0.0313 ± 0.0029
Black tea	0.0288 ± 0.0024	0.0313 ± 0.0026	0.0325 ± 0.0028	0.0319 ± 0.0029	0.0304 ± 0.0032
Wine	0.0295 ± 0.0020	0.0310 ± 0.0027	0.0315 ± 0.0024	0.0317 ± 0.0029	0.0319 ± 0.0021
Mean Ra values (± standard deviations) and statistical analysis of the tested universal composites at 21 days					
Artificial saliva	0.0338 ± 0.014^aA^	0.0381 ± 0.0033^aA^	0.0350 ± 0.0024^aA^	0.0365 ± 0.0012^aA^	0.0360 ± 0.0026^aA^
Coffee	0.0565 ± 0.080^bA^	0.0560 ± 0.0075^bA^	0.0617 ± 0.0066^bA^	0.0566 ± 0.0044^bA^	0.0677 ± 0.0028^bB^
Black tea	0.0596 ± 0.039^bA^	0.0654 ± 0.0088^cA^	0.0570 ± 0.0044^bA^	0.0575 ± 0.0042^bA^	0.0821 ± 0.0067^cB^
Wine	0.0579 ± 0.084^bABC^	0.0651 ± 0.0055^cC^	0.0612 ± 0.0036^bAC^	0.0544 ± 0.0049^bAB^	0.0518 ± 0.0048^ dB^

Different capital superscript letters indicate significant differences between different universal composites for each discoloring solution while different superscript letters indicate differences between discoloring solutions within each composite (*p* < 0.05)

After 21-days, there were no statistically significant differences between the composites stored in artificial saliva. In coffee and black tea, Estelite Asteria presented the highest Ra (*p* < 0.05), whereas there were no significant differences among the other composites (*p* > 0.05). In wine, the highest surface roughness was obtained with Filtek Universal (*p* < 0.05) which was not statistically different from that of G-aenial A’chord (*p* > 0.05). No statistically significant differences were observed among Estelite Asteria, G-aenial Injectable, and Filtek Ultimate (*p* > 0.05).

Coffee, black tea, and wine resulted in higher surface roughness than artificial saliva at 21-days. In contrast, coffee, black tea, and wine caused statistically similar surface roughening for Filtek Ultimate, G-aenial A’chord, and G-aenial Injectable (*p* > 0.05) whereas black tea resulted in a significantly higher Ra than coffee for Filtek Universal and Estelite Asteria (*p* < 0.05).

Regarding the correlation between surface roughness and color stability, there was a positive correlation in all the tested universal composites (*p* = 0.0001).

## Discussion

Meta-analyses [32, 33], systematic reviews [34, 35], and retrospective clinical evaluations [36] of anterior composite restorations revealed fractures, discoloration, and increase in surface roughness as the most common reasons for failure. Recently, the use of highly filled flowable universal composites favored by the advent of the injection moulding technique for different indications on the anterior and posterior region has been increasingly exploited [37]. Results of *in-vitro* research showed that they can be a valid alternative to paste-type universal composites in occlusal areas due to their similar flexural properties [38], surface roughness, and wear values, especially when they are overcured [37]. However, concerning discoloration and surface roughness, a comprehensive review of the existing literature does not address the full impact of different beverages on universal composites with different viscosities. Hence, this study aimed to evaluate the color stability, surface roughness, and surface morphology of nano-fill and nano-hybrid universal composites with different viscosities subjected to discoloring solutions, coffee, black tea, red wine, and artificial saliva as the control solution over a period of 21-days.

Discoloration of composite restorations is affected by the exposure period to discoloring agents [39]. However, in *in-vitro* studies, long-term storage in discoloring solutions is not preferred because brushing cannot be mimicked, and the residue of the solution cannot be removed from the surface [40, 41]. Therefore, in this study, the specimens were stored in discoloring solutions for 6 h a day, followed by brushing and storage in artificial saliva for 18 h for 21-days. Manufacturers indicate that consumers drink a cup of coffee for approximately 15 min and consume around 3 cups daily. When color stability was assessed in *in-vitro* tests using coffee as the discoloration solution, this application regimen was applied [41, 42]. Therefore, considering the total application period of the discoloring solutions in this study, it seems equivalent to approximately 3 years of clinical service.

Color evaluations in this study were performed with a spectrophotometer using the CIEDE2000 color evaluation system to investigate the susceptibility of the tested universal composites to discoloration [43, 44]. In several studies, ΔE 3.3 is considered the critical threshold value, which means that the color difference can be noticed by the naked eye, and it is clinically unacceptable. However, the threshold value for ∆E_00_ has been evaluated as 2.25 [45]. To increase the sensitivity and decrease the probability of missing clinically visible differences, lower values were established in a recent study by Paravina et al. [29]. ∆E_00_ < 0.8 is considered clinically perceptible; between 0.8 and 1.8 is clinically perceptible but acceptable; and beyond 1.8 is clinically unacceptable. All the tested universal composites reached the critical threshold value for ∆E_00_ in black tea, coffee, and wine at 7-days, and the ∆E_00_ gradually increased at the 14 and 21-day evaluation periods (Table 3). Regardless of the type of universal composite, statistically significant differences were observed between the discoloring solutions (Table 3; *p* < 0.001). Storage in artificial saliva resulted in the lowest ∆E_00_ for all the tested universal composites at all evaluation periods, and color change could only be noticed upon close inspection (Table 3), while red wine showed the highest color change among all the other discoloring solutions at 7 and 21-days evaluation periods (*p* < 0.001). Compared to artificial saliva, coffee, and black tea also resulted in clinically unacceptable color changes for all the tested universal composites at 7, 14, and 21-days of evaluation. Therefore, the first null hypothesis was rejected.

Coffee has been cited as one of the most discoloring solutions for resin composites [46]. In this study, universal composites stored in coffee underwent a lower color change than those stored in black tea or red wine. The alcohol and tannins in red wine cause a higher discoloration rate of the composites than other discoloring agents [23, 47]. The red wine used in this study contained 13% alcohol. The ability of ethanol to remove monomers, oligomers, and linear polymers from the surface has been reported to soften the composite surface. This decrease in surface hardness may increase the penetration of red wine pigments and cause further color changes [48, 49]. However, it has also been stated that alcohol can be inefficient in eliminating the polymer in the subsurface of resin composites due to the presence of water on this subsurface. Furthermore, surface breakdown by alcohol in a linear polymer may be more selective than in a cross-linked structure [50]. According to Satou et al. [51], the discoloration of resin composites can be caused by the interaction of hydrophilic unreacted components in the resin composite initiators with the hydrophilic pigments in red wine. In agreement with Ardu et al. [48] and Zajkani et al.’s [52] findings, black tea showed a considerably higher color change than artificial saliva but a lower color change than red wine in our study. In contrast to our findings, Ceci et al. [53] showed more discoloration with coffee than red wine. However, they dipped the specimens in cola solution first, followed by red wine and coffee, which could have affected the outcome. Similar to the results of our study, Chowdry et al. [54] showed that specimens exposed to black tea had a higher color change than coffee and coke, and artificial saliva resulted in the least color change. Their study also showed that the specimens exposed to black tea had the highest color change on the 14th and 28th-days of the experiment (ΔE values > 3.3), followed by coffee and coke. Regarding the ∆E_00_ values in our study, both the black tea and coffee groups reached the critical threshold value on the 7th day evaluation. However, the difference between these two discoloring solutions became significant at the 14 and 21-day evaluation periods, which also corresponded to the results of Satou et al. [51]**.** Black tea and coffee have yellow colorants with distinct polarities. Lower-polarity components, such as those in coffee, are eluted later, whereas higher-polarity components, such as those in black tea, are eluted earlier [12, 55]. This phenomenon may explain the results of our study that black tea resulted in higher discoloration than coffee for all the tested universal composites on days 14 and 21.

Bisphenol A-glycidyl methacrylate (Bis-GMA)-based resin matrix presents higher water sorption owing to its hydrophilicity, resulting in higher staining susceptibility compared to other methacrylate monomers, such as urethane dimethacrylate (UDMA). When Bis-GMA resins are combined with triethylene glycol dimethacrylate (TEGDMA) to manage viscosity, water uptake increases, making them more susceptible to increased staining [56, 57]. G-aenial A’chord and G-aenial Injectable do not contain Bis-GMA, whereas Estelite Asteria and Filtek Ultimate Universal composites contain Bis-GMA in their structure (Table 1). Highly filled flowable G-aenial Injectable showed statistically the lowest color change concerning red wine, coffee, and black tea on the 21st day of the evaluation period among all the tested universal composites (except the Filtek Ultimate black tea and wine groups), which was consistent with the results of Gawriołek, Maria, et al. [58]. One possible reason might be the lack of Bis-GMA in its composition, even though it contains TEGDMA, which has been reported to affect the extent of post-irradiation polymerization. As TEGDMA increases, the amount of post-irradiation polymerization decreases because the monomer generates a higher initial conversion, which helps improve the color stability [59]. Therefore, the low staining susceptibility of G-aenial Injectable may be related to the post-irradiation polymerization effect of TEGDMA and the absence of Bis-GMA. On the other hand, G-aenial A’chord also does not contain Bis-GMA and shares the Bis-MPEPP monomer with the same full-coverage silane coating (FSC) with G-aenial Injectable; however, it resulted in significantly higher color change than G-aenial Injectable on the 21st day of the evaluation period for all the tested discoloring solutions. This result can be attributed to the lack of TEGDMA, its post-irradiation polymerization effect, and the differences in the inorganic fillers of these materials (Table 1). G-aenial Injectable consists of 150 nm barium fillers, while G-aenial A’chord has HPC filler technology, an inorganic filler of 300 nm barium glass, and 16 nm fumed silica, which is pulverized to 10 µm [60, 61]. According to these results, the second hypothesis was also rejected.

**Fig. 1:**
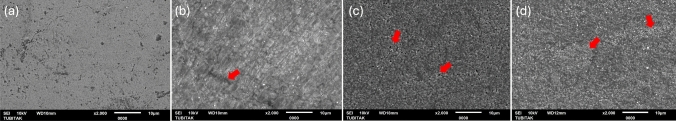
×2000 magnification SEM images of G-aenial Achord **a** baseline. **b** 21-day discolored in black tea. Slight erosion in the resin matrix is evident (red arrow). **c** 21-day discolored in coffee. Debonding of fillers leaving voids on the surface are present (red arrows). **d** 21-day discolored in red wine. Erosion in the resin matrix, protrusion of the fillers and some filler debondings can be seen (red arrows)

**Fig. 2:**
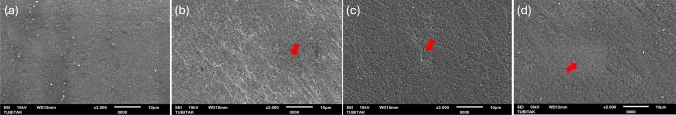
×2000 magnification SEM images of G-aenial Injectable. **a** baseline. **b** 21-day discolored in black tea. **c** 21-day discolored in coffee **d** 21-day discolored in red wine. While there are no filler debondings, some alterations in the resin matrix are present with all the discoloring beverages at 21-day evaluation

Consistent with the color stability data, the surface texture of the G-aenial A’chord presented differences in SEM evaluations compared to G-aenial Injectable. G-aenial A’chord presented some filler debondings, leaving voids on the surface in coffee (Fig. 1c) and erosion of the resin matrix in red wine at the 21-days evaluation (Fig. 1d), while no filler debondings were seen in G-aenial Injectable (Fig. 2) and although some alterations in the resin matrix was present, a smoother surface was evident after storage in red wine (Fig. 2d), which also corresponds well with the significantly higher surface roughness of G-aenial A’chord in red wine (Fig. 1d). Filtek Ultimate resulted in significantly lower color change than Filtek Universal. The low staining susceptibility of Filtek Ultimate may be related to the post-irradiation polymerization effect of TEGDMA. Additionally, Bis-EMA, a highly hydrophobic monomer [56], and Bis-EMA/TEGDMA, as base monomer formulations, have been shown to reduce water sorption and solubility compared to BisGMA/TEGDMA or UDMA/TEGDMA formulations, along with better color stability [62]. When Bis-GMA resins are combined with TEGDMA to manage viscosity, water uptake increases, resulting in increased discoloration [56, 62]. A high filler load has been shown to reduce the color change of resin composites; however, in this study, the universal composite Estelite Asteria had the highest filler load (82% wt; 71% vol), resulting in a significantly higher color change than G-aenial Injectable and Filtek Ultimate. Fillers that are much harder than the resin matrix may cause prominent matrix abrasion during polishing, which may explain the higher discoloration results with this material because it contains silica/zirconia fillers [63]. Concerning Estelite Asteria, consistent with the data of Ersöz B et al., this supra-nanofil universal composite resulted in higher discoloration in coffee compared to a nanohybrid composite [64].

**Fig. 3:**
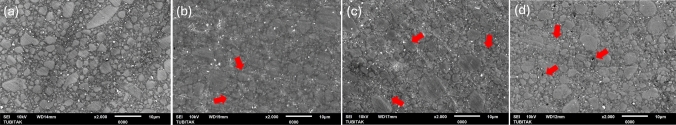
×2000 magnification SEM images of Filtek Universal **a** baseline. Abrasion in the resin matrix, protrusion of the fillers along with some superficial abrasion marks on the filler surfaces are evident (red arrows). **b** 21-day discolored in black tea **c** 21-day discolored in coffee **d** 21-day discolored in red wine. Slight erosion in the resin matrix and some filler debonding can be seen (red arrows)

**Fig. 4:**
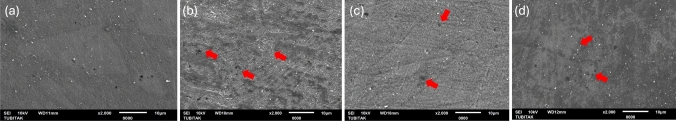
×2000 magnification SEM images of Estelite Asteria **a** baseline. Debonding of the inorganic fillers is prominent in baseline and after exposure to all the discoloring beverages (red arrows). **b** 21-day discolored in black tea. Alterations in the resin matrix can be seen **c** 21-day discolored in coffee. **d** 21-day discolored in red wine

**Fig. 5:**
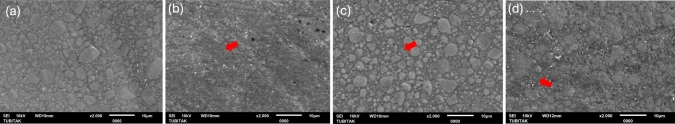
×2000 magnification SEM images of Filtek Ultimate **a** baseline. Abrasion in the resin matrix and protrusion of the fillers are evident. **b** 21-day discolored in black tea. Alterations in the resin matrix with some filler debondings can be seen. **c** 21-day discolored in coffee **d** 21-day discolored in red wine. Slight erosion in the resin matrix is evident (red arrow)

Surface alterations, such as superficial abrasion marks on the filler surfaces of Filtek Universal (Fig. 3) and filler debondings in Estelite Asteria (Fig. 4), were observed even at the baseline SEM evaluations, indicating different effects of the polishing systems on the resin composite surface topography. All the tested universal composites in this study revealed significantly higher Ra values after exposure to discoloring agents(*p* < 0.001). However, except for Filtek Universal and Estelite Asteria, no significant differences in Ra were observed among red wine, black tea, and coffee for each composite (*p* > 0.05). Similar to our results, Chowdry et al. [54] showed that nanohybrid resin composites resulted in higher surface roughness in black tea and coffee, which did not significantly differ from each other, and artificial saliva showed the lowest surface roughness. Therefore, the third hypothesis was rejected. In terms of black tea and coffee, Estelite Asteria presented the highest surface roughness among all the tested universal composites (*p* < 0.05), while there were no statistically significant differences between Filtek Ultimate, Filtek Universal, G-aenial A’chord, and G-aenial Injectable at 21-days of evaluation (*p* > 0.001). Hence, the fourth hypothesis was also rejected. For red wine, Filtek Universal and G-eanial A’chord had the highest Ra values. Consistent with the Ra data, SEM evaluations of the G-eanial A’chord revealed erosion in the resin matrix and protrusion of the fillers (Fig. 1), while SEM of Filtek Universal showed erosion in the resin matrix along with some filler debonding (Fig. 3). Additionally, SEM evaluation of Filtek Ultimate showed alterations in the resin matrix and debonding of the filler particles in black tea after 21-days of evaluation (Fig. 5b).

According to the surface roughness results, none of the tested universal composites reached the threshold value (0.2 µm) for the increase in plaque accumulation [65–67]. All the tested universal composites presented low Ra values at baseline and after exposure to different discoloring solutions at the 21-day evaluation, most probably because of the meticulous polishing procedures of the specimens with descending degrees of abrasive polishing wheels and diamond polishing pastes of up to 0.5 µm. A higher filler content is expected to protect the resin matrix from excessive abrasion, resulting in smoother surfaces [68]. However, as mentioned earlier, fillers (silica/zirconia) that are much harder than the resin matrix may cause prominent matrix abrasion during polishing [38]. The abrasion of the softer resin matrix may result in a lack of support for the fillers, leading to further filler debonding and roughening of the surface [69]. As shown in the SEM, debonding of the inorganic fillers was prominent with Estelite Asteria (Fig. 4), and surface alterations, such as superficial abrasion marks, on the filler surfaces of Filtek Universal (Fig. 3a) at the baseline SEM evaluations, resulted in a further increase in surface roughness after immersion in discoloring solutions after 21-days [61] (Fig. 3b, c, d).

One of the limitations of this study was the immersion protocol of the tested universal composites in discoloring solutions, which did not apply an actual brushing regimen with toothpaste. Another limitation was the duration of the discoloration protocol; extended evaluation periods may provide more insight into the discoloration and surface roughness patterns of the universal composites. Furthermore, optical profilometers offer more detailed information about the surface topography of resin composites, and the evaluation of surface roughness with a contact-type profilometer may not be sufficient to determine the effects of different discoloring solutions on the surface roughness of universal composites. Therefore, further studies with extended evaluation periods and more advanced methods would be beneficial.

## Conclusions

Within the limitations of this in vitro study, the following conclusions were drawn:

- All the tested universal composites revealed clinically unacceptable color change (∆E_00_ > 1.8) and increased surface roughness after exposure to discoloring solutions; however, the increase in surface roughness did not reach the critical threshold value for plaque accumulation.
- Red wine resulted in the highest color change for all the tested universal composites followed by black tea and coffee.
- Highly filled flowable universal composite showed the highest color stability in coffee, red wine, and black tea.

### Clinical relevance

Universal composites with different viscosities may reveal clinically unacceptable color change and increased surface roughness after exposure to coffee, black tea, and red wine. Regarding the color stability and surface roughness highly filled flowable universal composite can be an alternative to paste-type universal composites.
